# Surface Microstructure Engineering for Enhancing Li-Ion Diffusion and Structure Stability of Ni-Rich Cathode Materials

**DOI:** 10.3390/nano15151144

**Published:** 2025-07-24

**Authors:** Huanming Zhuo, Shuangshuang Zhao, Ruijie Xu, Lu Zhou, Ye Li, Yuehuan Peng, Xuelong Rao, Yuqiang Tao, Xing Ou

**Affiliations:** 1 School of Chemistry and Chemical Engineering, University of South China, Hengyang 421001, China; 2 School of Metallurgy and Environment, Central South University, Changsha 410083, China

**Keywords:** lithium-ion battery, Ni-rich cathodes, heterojunction construction, diffusion kinetics, interfacial stability

## Abstract

Surface microstructure of grains vastly decides the electrochemical performance of nickel-rich oxide cathodes, which can improve their interfacial kinetics and structural stability to realize their further popularization. Herein, taking the representative LiNi_0.8_Co_0.15_Al_0.05_O_2_ (NCA) materials as an example, a surface heterojunction structure construction strategy to enhance the interface characteristics of high-nickel materials by introducing interfacial ZnO sites has been designed (NCA@ZnO). Impressively, this heterointerface creates a strong built-in electric field, which significantly improves electron/Li-ion diffusion kinetics. Concurrently, the ZnO layer acts as an effective physical barrier against electrolyte corrosion, notably suppressing interfacial parasitic reactions and ultimately optimizing the structure stability of NCA@ZnO. Benefiting from synchronous optimization of interface stability and kinetics, NCA@ZnO exhibits advanced cycling performance with the capacity retention of 83.7% after 160 cycles at a superhigh rate of 3 C during 3.0–4.5 V. The prominent electrochemical performance effectively confirms that the surface structure design provides a critical approach toward obtaining high-performance cathode materials with enhanced long-cycling stability.

## 1. Introduction

Lithium-ion batteries, distinguished by their exceptional energy density and economic viability, have achieved widespread adoption across electric vehicles (EVs), portable electronics, and grid-scale energy storage systems [1,2,3,4]. The electrochemical performance of these batteries, particularly critical for EV applications, fundamentally dictates vehicle autonomy and market competitiveness, with cathode materials constituting the most pivotal determinant [5]. Within this context, nickel-rich LiNi_x_Co_y_Al_1−x−y_O_2_ (x ≥ 0.8) layered oxides have emerged as a preeminent cathode candidate in contemporary battery research, owing to their superior specific capacity and favorable cost-to-performance ratio [6,7,8,9,10,11,12,13,14].

Unfortunately, the further application of LiNi_x_Co_y_Al_1−x−y_O_2_ materials was limited by the serious capacity decay [15,16,17,18,19]. Firstly, the poor stability of LiNi_x_Co_y_Al_1−x−y_O_2_ resulted in the interfacial parasitic reactions due to the presence of Ni^4+^ at a highly charged state, including the decomposition of electrolyte and the escape of lattice oxygen. Meanwhile, the reduced ions of Ni^2+^ easily occupy the surrounding Li^+^ sites, which can induce the formation of cation mixing and rock-salt structure, thus leading to depressed Li^+^ diffusion and the loss of irreversible capacity [20]. In addition, the accumulated internal stress induced by the harmful phase transitions from H2 to H3 can create microcracks, which can accelerate the performance deterioration. The reason is that the electrolyte can penetrate into the interior of the material through microcracks, which facilitate the further depletion of electrolyte, resulting in aggravation of capacity fading [21]. Thus, it is urgently necessary to exploit an effective modification strategy to overcome the aforementioned challenges and promote the large-scale application of high-nickel materials.

Coating has been served as a commonly used and highly effectively method for reinforcing the interfacial stability of Ni-rich materials by restraining the interfacial parasitic reactions [22,23,24,25]. Su et al. reported the Ni-rich layered cathodes with La_4_NiLiO_8_-modified surface, which improves their structure durability by restraining the formation of interfacial oxygen vacancies and rock-salt structure [26]. Meanwhile, the Young’s modulus of the Ni-rich materials can also be effectively enhanced by introducing a LiNbO_3_-protective layer, thereby strengthening the cycling stability of the Ni-rich materials, as proved by Zuo et al. [27]. Notably, introducing some metal oxide semiconductor materials with relatively high electrical conductivity can help the construction of a surface heterostructure between the target and coated materials. This not only enables the formation of the built-in electric field to facilitate the Li^+^ diffusion kinetics but also forms a protective barrier to prevent the occurrence of heterogeneous interface side reactions, thereby promoting the high-rate cycling stability of the Ni-rich materials [28]. As illustrated by Ran et al. [29], the designed surface heterostructure delivers the expected features to restrain the interfacial side reaction and improve the Li^+^ diffusion rate, thereby enhancing the cycling stability of prepared cathode materials. Noteworthily, as the semiconductor phase, ZnO material possesses the excellent properties of great electrical conductivity and chemical stability compared with Al_2_O_3_ and ZrO_2_, enabling it to simultaneously enhance the reaction kinetics and stability of Ni-rich material [28,30,31].

Herein, the semiconductor material of ZnO was employed as the protective layer to reinforce the interfacial stability and weaken Li^+^ insertion energy barrier at surface of nickel-rich layered cathode LiNi_0.8_Co_0.15_Al_0.05_O_2_ (NCA) with the following advantages: Firstly, the construction of ZnO protective barrier can effectively suppress the erosion of electrode materials by electrolyte, which is conducive to heighten the cycling stability and reduce the interfacial side reaction. Secondly, the interface between ZnO and NCA materials can form an electric field due to the difference in work function of the two materials, which can greatly accelerate the process of Li^+^ desolvation and insertion. Thus, the ZnO-modified NCA electrode materials deliver the enhanced cycling stability at an ultrahigh rate of 3 C during 3.0–4.5 V with the capacity retention rate of 83.7% after 160 cycles. Meanwhile, the target materials also show reinforced cycling performance in full cells at 3.0–4.2 V.

## 2. Materials and Methods

### 2.1. Materials and Synthesis

#### 2.1.1. Synthesis of ZIF-8

Zn(NO_3_)_2_·6H_2_O (99.0%, Aladdin, Shanghai, China) and 2-methylimidazole (98%, Macklin, Shanghai, China) with a molar ratio of 1:4 were dissolved in 100 mL of methanol, and the mixed solution reacted at room temperature for 24 h. Subsequently, the resulting white ZIF-8 precipitate was collected after centrifugation and drying at 70 °C for 48 h.

#### 2.1.2. Synthesis of NCA and NCA@ZnO

The NCA@ZnO composite was synthesized via a solid-state mixing method. Initially, a commercial Ni_0.8_Co_0.15_Al_0.05_(OH)_2_ precursor (Zhichuan, Haian, China) was homogeneously mixed with LiOH·H_2_O (Aladdin, 99%) at a molar ratio of 1:1.07. The mixture was first thermally treated at 450 °C for 5 h, followed by calcination at 760 °C for 12 h in an oxygen atmosphere with a heating rate of 10 min^−1^ to obtain spherical-structured lithium nickel cobalt aluminum oxide (NCA). The as-prepared NCA was then mixed with ZIF-8 at a molar ratio of 100:1. The mixture was heated to 600 °C at a rate of 5 °C min^−1^ in air and maintained for 2 h, during which ZIF-8 was pyrolyzed to form ZnO, resulting in the final ZnO-coated NCA@ZnO composite.

### 2.2. Characterizations

The crystal structure and phases were determined by using an X-ray diffraction (XRD) (Rigaku X-ray diffractometer, Tokyo, Japan, Cu Kα, λ = 1.5418 Å) with a scanning rate of 10° min^−1^. The morphology characterization of all the samples was conducted by using the scanning electron microscopy (SEM, Nova NanoSEM-23, Hillsboro, OR, USA). The results of microscopic structure and elemental mapping were obtained in high-resolution transmission electron microscopy (HRTEM) and energy-dispersive X-ray spectroscopy (EDS) (Tecnai G2 F20 S-TWIN, Tokyo, Japan), respectively. Surface element information was obtained using X-ray photoelectron spectroscopy (XPS, Kratos Model XSAM800, Singapore).

### 2.3. Electrochemical Tests

The electrochemical performance of the sample was obtained through CR2032 coin-type cells. The work electrode includes the 10 wt% polyvinylidene fluoride (PVDF), 10 wt% acetylene black, and 80 wt% as-prepared materials. The diameter of the work electrode is φ12 mm, and its loading weight is about 2.5 mg cm^−2^. The cells are assembled in an Ar-filled glove box (H_2_O < 0.1 ppm, O_2_ < 0.1 ppm). The half-cells are composed of lithium foil as the counter/reference electrode, the prepared work electrode, a solution of 1.0 M LiPF_6_ in dimethyl carbonate (DMC)-ethylene carbonate (EC)-ethyl methyl carbonate (EMC) (1:1:1 by volume) as the electrolyte and polypropylene as the diaphragm (Celgard 2400, Charlotte, NC, USA). The full-cells were fabricated by encapsulating NCA-based cathodes, graphite anodes, the aforementioned electrolyte, and separators within aluminum laminate film packaging. The charged/discharged curves were performed in a battery test system (NEWARE) at various rates in 3.0–4.5 V for half-cells and 3.0–4.2 V for full-cells. All the cells have been activated at 0.1C for the first three cycles. Electrochemical impedance spectroscopy (EIS) was performed on the electrochemical workstation CHI760E (Beijing, China) over a frequency range of 0.01 Hz to 100 kHz with an amplitude of 5 mV.

## 3. Results

### 3.1. Characterization of Materials

**Figure 1:**
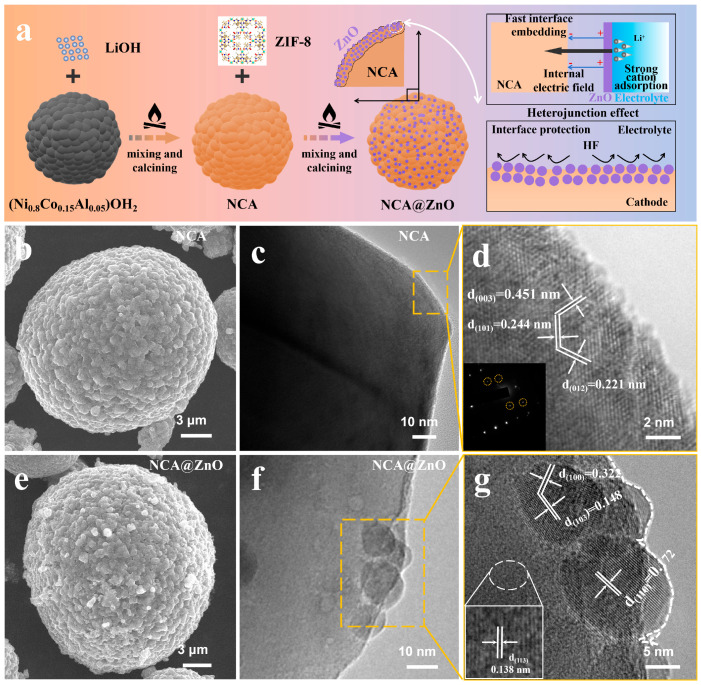
(**a**) The schematic illustration of production process of the two samples and the modification mechanism. The SEM, TEM, and HRTEM for NCA (**b**–**d**) and NCA@ZnO (**e**–**g**).

The facile fabrication process of the NCA@ZnO composite has been schematically illustrated in Figure 1a; the design of the ZnO protective layer is highly anticipated to alleviate interface corrosion and enhance the kinetics of Li^+^ insertion at the interface between electrode and electrolyte. The scanning electron microscope (SEM) images of NCA and NCA@ZnO, displayed in Figure 1b,e, reveal that both materials exhibit a spherical morphology composed of nanoparticles, with diameters of about 20 µm [3,32]. Meanwhile, the similar framework structure demonstrates that the introduction of ZnO delivers an almost negligible effect on the morphology of NCA. In detail, compared with the surface state of NCA (Figure 1b), the NCA@ZnO materials deliver a coarser surface texture (Figure 1e), which is induced by heterogeneous nano-deposits, preliminarily demonstrating the successful construction of a ZnO-coating layer at the surface of NCA. To further confirm the structure and interface variations resulting from introducing ZnO, transmission electron microscopy (TEM) was employed in Figure 1. The results reveal that NCA material delivers a smooth surface (Figure 1c), while the surface of NCA@ZnO material expresses obvious deposition of nanoparticles (Figure 1f), effectively proving the successful modification of NCA@ZnO material by using a heterogeneous phase. The representative layered structure of NCA has been confirmed by utilizing high-resolution TEM (HRTEM) as shown in Figure 1d, where the clearly lattice fringes with 0.221 nm, 0.244 nm, and 0.451 nm belonging to (012), (101), and (003) of the R-3m structure, respectively, have been analyzed.

Additionally, small spherical channels are unevenly distributed on the surface of the aggregated particles, serving as connections between the ionic interface and the electrolyte. This structure facilitates electrochemical reactions and enhances kinetics by promoting efficient electrolyte penetration and ensuring short diffusion distances [1]. In addition, the crystal structure of NCA@ZnO in the bulk delivers a similar structural characteristic, which corresponds well with the R-3m space groups (Figure 1g). Meanwhile, at the surface of NCA@ZnO, the clear coating layer can be confirmed, and the planes with the interplanar spacing of 0.172 nm, 0.148 nm, and 0.322 nm are in accord with (110), (103), and (100) of ZnO, demonstrating the successful construction of the designed structure. Meanwhile, the elemental mapping results (Figures S4 and S5 in Supporting Information) further evidence the above conclusion. Predictably, the interface between NCA and ZnO can form a heterojunction structure and protective barrier, which can generate a built-in electric field and severely restrain the side reaction of electrolyte, thus reinforcing the Li^+^ insertion kinetics and interface structure stability, respectively. This design will effectively ensure the material achieves enhanced cycling stability under high-rate and high-voltage conditions.

**Figure 2:**
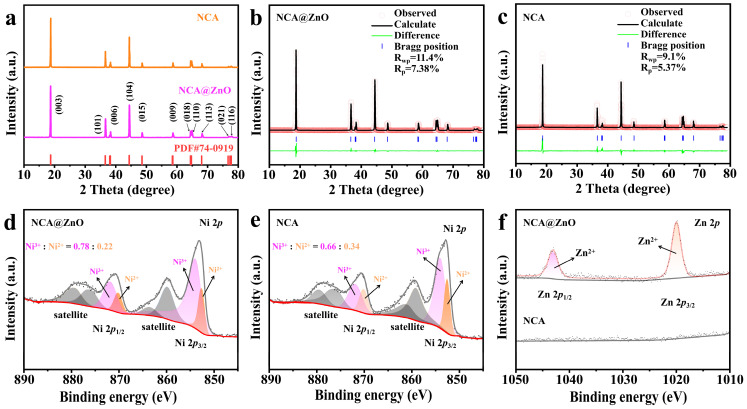
(**a**) The XRD of NCA and NCA@ZnO. XRD Rietveld refinement results of the NCA@ZnO (**b**) and NCA (**c**). The XPS of Ni 2*p* for NCA@ZnO (**d**) and NCA (**e**). (**f**) The XPS of Zn 2*p* for NCA@ZnO and NCA.

The crystalline phase characteristics have been further investigated by using XRD, as shown in Figure 2a. All diffraction patterns of the two samples are well indexed into the layered α-NaFeO_2_ structure with R-3m space groups, and there is no obvious variation between NCA@ZnO and NCA materials, demonstrating negligible influence of introducing ZnO into the surface of NCA. Meanwhile, both samples possess a greatly layered structure confirmed by the higher values of *I*_(003)_/*I*_(104)_ than 1.2 [33]. This result fully illustrates that this coated modification strategy does not cause the phase transition and crystal structure evolution in NCA@ZnO, which may be due to the relatively low content of the introduced ZnO. Furthermore, the crystal structure has been deeply analyzed by Rietveld refinement of XRD results (Figure 2b,c), which delivers satisfactory reliability because of the low values of *R*_p_ and *R*_wp_. It demonstrates that the contents of Ni^2+^ occupation within the Li sites (Table S1) for NCA@ZnO (3.8%) are less than that of NCA electrode materials (4.2%), proving the decreased Li^+^/Ni^2+^ mixing and enhanced orderliness of the layered structure resulting from the modification of ZnO on the surface of NCA [34,35,36]. Meanwhile, the bigger c/a value in NCA@ZnO (4.945037) compared with that of NCA (4.936670) can further illustrate the well-layered structure characteristics of the designed materials. This result indicates that the NCA@ZnO material will exhibit excellent lithium storage performance.

To investigate the evolution of surface element composition and valence state after introducing ZnO, the X-ray photoelectron spectroscopy (XPS) was performed (Figure S6). Figure 2d,e compares the Ni 2*p* spectra of the modified NCA@ZnO and pristine NCA samples, and XPS analysis reveals significant modulation of Ni oxidation states in ZnO-coated NCA compared to pristine NCA. For Ni 2*p* spectra of the two samples, the Ni 2*p*_3/2_ spectrum exhibits characteristic peaks at 852.5 eV (Ni^2+^) and 854.0 eV (Ni^3+^), while the Ni 2*p*_1/2_ spectrum shows peaks at 870.1 eV (Ni^2+^) and 871.8 eV (Ni^3+^). Meanwhile, it can be inferred that the content of Ni^2+^ in NCA (22%) is higher compared with that of NCA@ZnO (34%), which is in agreement with the tendency of XRD analysis, demonstrating the enhanced cation arrangement orderliness in modified NCA@ZnO materials. The increased Ni^3+^ population in NCA@ZnO suggests modified surface redox activity, potentially enhancing rate capability [37,38,39,40]. Moreover, as shown in Figure 2f, NCA@ZnO exhibits two distinct Zn 2*p* peaks with binding energies of 1020.5 eV (Zn 2*p*_3/2_) and 1043.6 eV (Zn 2*p*_1/2_), respectively, which are absent in the NCA sample. This observation aligns with the results obtained from HRTEM analysis, further confirming the successful introduction of ZnO. Collectively, the XRD, SEM, TEM, and XPS results demonstrate the integrity and accuracy of the surface interface structure design, and the successful incorporation of ZnO on the NCA surface contributes to enhancing the electrochemical properties of NCA@ZnO.

### 3.2. Electrochemical Properties

**Figure 3:**
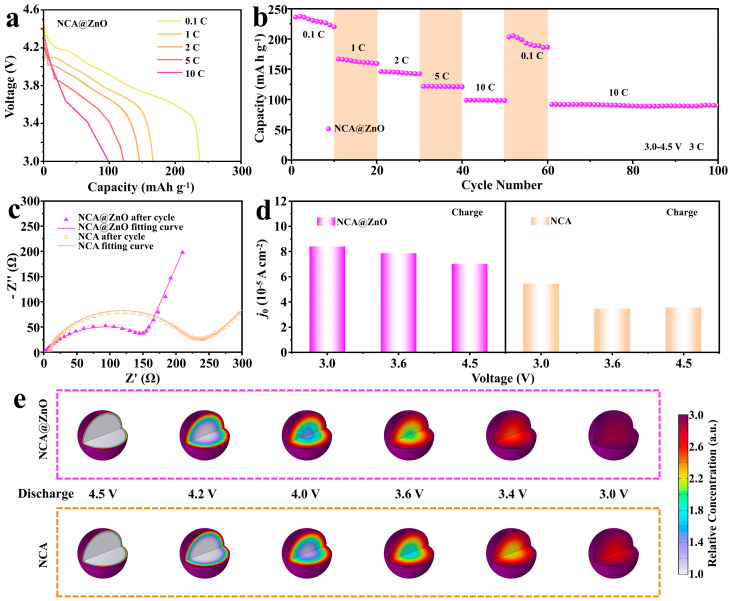
The discharging curves (**a**) and rate performance (**b**) at different rates of NCA@ZnO. (**c**) The EIS results of NCA@ZnO and NCA after cycling. (**d**) R_ct_-derived exchange current density for the two samples. (**e**) The Li^+^ concentration distribution of the two samples was calculated by COMSOL during discharging process.

To evaluate the impact of ZnO modification on the electrochemical properties of NCA materials, the NCA@ZnO and NCA electrodes were analyzed by using galvanostatic charge–discharge techniques within a potential range of 3.0–4.5 V. As the increase in current density (1 C = 180 mAh g^−1^), the discharged specific capacity of the two samples delivers the gradually decreased tendency (Figure 3a and Figure S7). In detail, the specific capacity of the NCA@ZnO cathode is 240.7 mAh g^−1^, 163.9 mAh g^−1^, 146.6 mAh g^−1^, and 122.3 mAh g^−1^ at current densities of 0.1 C, 1 C, 2 C, and 5 C, respectively (Figure 3b). Even increased to 10 C, it can still obtain the capacity of 99.4 mAh g^−1^. When the rate returns to 0.1 C, the NCA@ZnO electrode material delivers a similar capacity to its original value. More significantly, after it continued to react at a rate of 10 C for 40 cycles, it still maintains a considerable capacity of 91.9 mAh g^−1^, demonstrating the enhanced reaction kinetics and rate tolerance resulting from ZnO-coating modification. As a contrast, the specific capacities of the NCA material are 246.1 mAh g^−1^, 164.8 mAh g^−1^, 143.7 mAh g^−1^, 118.6 mAh g^−1^, 74.0 mAh g^−1^, and 198.3 mAh g^−1^ at current densities of 0.1 C, 1 C, 2 C, 5 C, 10 C, and 0.1 C in Figure S8, respectively. Obviously, the rate performance of NCA@ZnO surpasses that of NCA, demonstrating that the incorporation of ZnO significantly enhances the rate performance of the Ni-rich electrode materials [41,42].

The EIS test has been employed to verify the enhanced rate performance of NCA@ZnO materials, and the results have been fitted by using the equivalent circuit in Figure S9. The fitted Nyquist plots can yield all relevant impedance parameters, including the ohmic resistance at the intersection of curves and the X-axis, the charge transfer resistance at high frequency (*R*_ct_), and the Warburg resistance at low frequency. According to the analytical EIS results in Figure 3c and Figure S10 and Table S2, the results show significantly lower charge transfer resistances (*R*_ct_ = 97.5 Ω before cycling, *R*_ct_ = 186.5 Ω after cycling) and higher Li^+^ diffusion coefficient (5.17 × 10^−12^ cm^2^ s^−1^, after cycling) for NCA@ZnO than those (*R*_ct_ = 143.5 Ω before cycling, *R*_ct_ = 224.8 Ω after cycling, *D*_Li+_ = 2.01 × 10^−12^ cm^2^ s^−1^ after cycling) of NCA, which illustrates the enhanced reaction kinetics of the designed cathode. Thereof, this result effectively proves the enhanced rate performance of NCA@ZnO, which can give credit to the ZnO coating at the surface of target materials. To further validate our hypothesis, we conducted in situ EIS measurements for both NCA@ZnO and NCA (Figure S11). The exchange current density (*j*_0_, Figure 3d) can be calculated by the advanced treatment of *R*_ct_-values at various voltages based on the following equation: *j*_0_ = *RT*/*nFR*_ct_*A*. In this equation, *R* represents the gas constant, and the others are absolute temperature, the number of electrons during reaction, the Faraday’s constant, and the interfacial area of the electrode, separately. Notably, the *j*_0_-value is positively correlated with the Li^+^ insertion rate. Obviously, the designed NCA@ZnO exhibits a greater advantage in terms of interfacial Li^+^ transport compared with NCA, which provides additional evidence for substantiating our hypothesis. To further elucidate the distinct electrochemical kinetics between ZnO-coated and pristine NCA cathodes, we conducted the simulation of Li^+^ diffusion kinetics via the COMSOL Multiphysics 6.3 platform, establishing the ion transport model based on SEM results (Figure 3e). The computational results demonstrate that both systems initially exhibit the characteristic of lithium concentration gradient distribution during the discharging state. In detail, the Li^+^ content delivers a gradually reduced tendency from surface to core, and the homogeneity of Li^+^ distribution becomes increasingly uniform as the reaction progresses. According to the simulated results, unmodified NCA maintains this radial heterogeneity, displaying a signature of diffusion-limited transport mechanisms with the lithium-rich surface layers and lithium-depleted core regions. In striking contrast, the NCA@ZnO composite achieved bulk-phase homogeneity with rapid lithium redistribution, attaining near-equilibrium concentration profiles across both interfacial and bulk regions within the simulated time frame. These computational findings quantitatively confirm that ZnO interfacial engineering enhances lithium-ion transport kinetics. This numerical evidence aligns coherently with our crystallographic characterization and electrochemical performance metrics, collectively establishing a mechanistic framework that correlates surface modification strategies with enhanced electrode reaction dynamics through optimized ion diffusion pathways.

**Figure 4:**
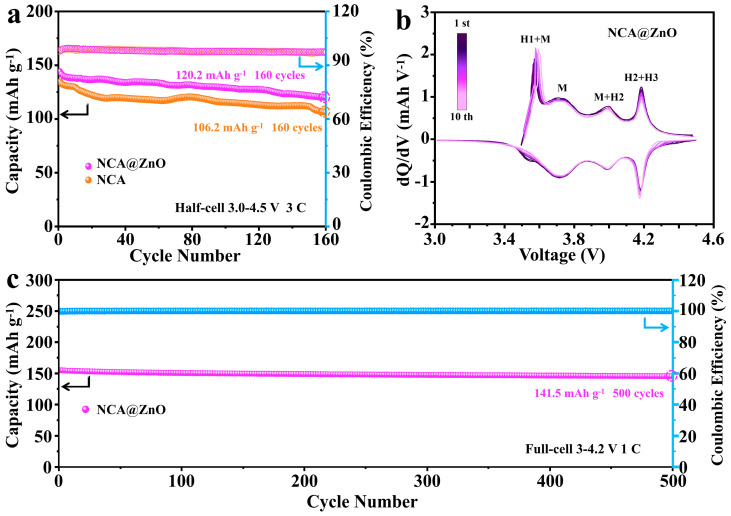
(**a**) The cycling performance at 3 C for the two samples. (**b**) The dQ/dV curves of NCA@ZnO with different cycling numbers. (**c**) The cycling performance of full-cell for NCA@ZnO at 1 C.

The long-term cycling stability of NCA@ZnO and NCA cathodes was further evaluated at a high rate of 3 C (Figure 4a). Notably, the NCA@ZnO cathode exhibited a stable discharge capacity of 120.2 mAh g^−1^ after 160 cycles, with a high capacity retention of 83.7% and a Coulombic efficiency (CE) close to 100%. In contrast, the NCA cathode delivered a significantly lower discharge capacity of 106.2 mAh g^−1^, with a capacity retention of only 78.9%. Meanwhile, the loss fading has also been visually confirmed by using charging–discharging curves as presented in Figure S12. The differential capacity (dQ/dV) profiles of NCA@ZnO and NCA were analyzed across multiple cycles to elucidate phase transition dynamics during charge–discharge processes and evaluate the structural stability, as shown in Figure 4b and Figure S13. Both samples displayed several peaks in their profiles, which represent the continuous phase transitions of H1 to M, M to H2, and H2 to H3. Thereof, the almost overlapping curves effectively demonstrate the excellent reversibility and cycle stability of NCA@ZnO. Additionally, the electrochemical performance of full cells has also demonstrated the effectiveness and practicality of ZnO-coating NCA materials, as shown in Figure 4c. The modified NCA@ZnO delivers the capacity of 141.5 mAh g^−1^ after 500 cycles at 1 C during 3.0–4.2 V and nearly 100% Coulombic efficiency, highlighting its superior long-cycle stability and practical significance.

## 4. Discussion

**Figure 5:**
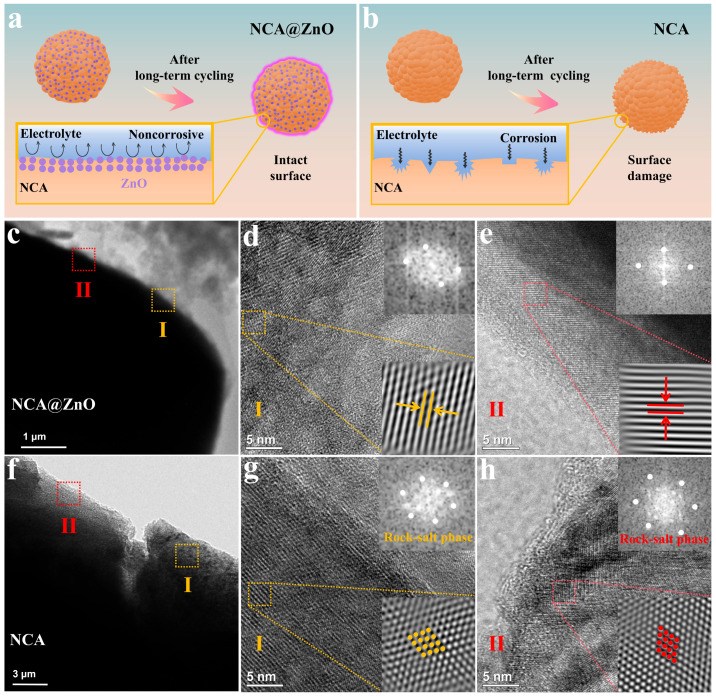
(**a**,**b**) Mechanism diagram of material stability after long-term cycling. (**c**,**f**) TEM and (**d**,**e**,**g**,**h**) HRTEM after NCA@ZnO and NCA cycles.

The intergranular crack resulting from the degradation of interfacial structure stability can lead to a further increase in capacity attenuation [43]. In addition, the harmful reaction between electrolyte and electrode materials can deteriorate the interfacial framework. Thus, enhancing the interfacial stability can effectively reinforce the cycling performance of Ni-rich materials. As shown in Figure 5a,b, the ZnO-coating layer on NCA@ZnO effectively suppresses parasitic reaction induced by HF corrosion from the electrolyte during cycling, thereby maintaining structural integrity. As expected, the interface optimization enables the NCA@ZnO cathode to preserve its original morphological configuration after prolonged cycling (Figure 5c). In contrast, the unmodified NCA counterpart exhibits severe structural degradation (Figure 5f) due to the unstable interface structure resulting from the chemical erosion of the electrolyte.

The interface structure stability of the constructed NCA@ZnO can be further demonstrated by utilizing the HRTEM test. After cycling, the NCA@ZnO sample still exhibits relatively clear lattice fringes with a layered structure in various surface areas (Figure 5d,e), whereas the surface of the cycled NCA shows significant rock-salt phase in the two selected areas (Figure 5g,h). Meanwhile, the Fourier transform (FFT) derived from HRTEM images by using Digital-Micrograph 3.6 software further confirms the presence of the rock-salt phase in NCA. Notably, the rock-salt phase is an electrochemically inert layer, which impedes lithium-ion diffusion kinetics. Thus, this result effectively demonstrates the obvious advantage of NCA@ZnO in terms of structure stability and reversibility, confirming that ZnO modification effectively inhibits the structure collapse of NCA@ZnO materials and improves their cycling stability.

## 5. Conclusions

In summary, the ZnO-modified NCA@ZnO materials were formed with the formation of an interfacial protective layer and heterostructure by using a hybrid sintering method in this work. The existence of a heterostructure generates the built-in electric field at the surface of NCA@ZnO, which weakens the Li^+^ insertion barrier, thereby improving its reaction kinetics. Moreover, the formed ZnO layer can serve as a protective barrier to restrain the corrosion induced by electrolyte, thereby strengthening the interfacial structure stability. Benefitting from the above effective modification, NCA@ZnO delivered a prominent capacity retention ratio of 83.7% after 160 cycles at 3 C during 3.0–4.5 V. These results demonstrate that integrating surface microstructure design by introducing hetero-phase can significantly enhance the electrochemical performance of high-nickel cathode materials. However, more effective strategies are still needed to reinforce the crystal structure and interface problems of Ni-rich layered oxide cathodes during long-term cycling at high voltage. Wishfully, this surface microstructure engineering can provide a promising development direction for high-performance lithium-ion battery electrode materials.

## Data Availability

The data that support the findings of this study are available upon request from the corresponding author.

## Supplementary Materials

The following supporting information can be downloaded at: https://www.mdpi.com/article/10.3390/nano15151144/s1; Figure S1: The XRD result of ZIF-8; Figure S2: The SEM result of ZIF-8; Figure S3: The TGA result of ZIF-8; Figure S4: The SEM elemental mapping of NCA@ZnO; Figure S5: The TEM elemental mapping of NCA@ZnO; Figure S6: XPS result of NCA@ZnO and NCA; Figure S7: The discharging curves of NCA at various rates; Figure S8: The rate performance of NCA; Figure S9: The equivalent circuit for fitting EIS curves of NCA and NCA@ZnO; Figure S10: The discharging/charging curves of NCA@ZnO (a) and NCA (b) at various cycles; Figure S11. In situ EIS in charge process for (a,b) NCA@ZnO and (c,d) NCA composites, respectively. Figure S12: The dQ/dV curves of NCA with different cycling numbers; Figure S13: The EIS results (c) of NCA@ZnO and NCA before cycling; Table S1. The refined lattice parameters, c/a and Li/Ni mixing results for NCA@ZnO; Table S2. The Rct results of NCA and NCA@ZnO in the equivalent circuit; Table S3. The Warburg (σ_ω_) results of NCA and NCA@ZnO before and after cycle; Table S4. The D_Li+_ results of NCA and NCA@ZnO before and after cycle.
