# Supplementary material for: Surface Microstructure Engineering for Enhancing Li-Ion Diffusion and Structure Stability of Ni-Rich Cathode Materials
Source: Nanomaterials (Basel). 2025 Jul 24;15(15):1144. doi: 10.3390/nano15151144 (PMC12348175; doi:10.3390/nano15151144)
Supplement: Supplementary file 1 [file nanomaterials-15-01144-s001.zip › nanomaterials-3784029-supplementary.pdf]

Surface Microstructure Engineering for Enhancing Li-Ion Diffusion and Structure Stability of Ni-Rich Cathode Materials

Huanming Zhuo [1,†], Shuangshuang Zhao [1,†], Ruijie Xu [1], Lu Zhou [1], Ye Li [1], Yuehuan Peng [1], Xuelong Rao [1], Yuqiang Tao [1,*] and Xing Ou [2]

[1] School of Chemistry and Chemical Engineering, University of South China, Hengyang 421001, China
[2] School of Metallurgy and Environment, Central South University, Changsha 410083, China
* Correspondence: taoyuqiang@usc.edu.cn
† These authors contributed equally to this work.

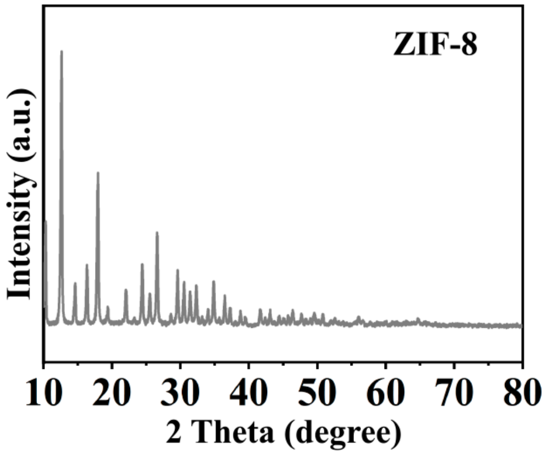

Figure S1. The XRD of ZIF-8.

The successful preparation of ZIF-8 has been confirmed by using XRD (**Figure S1**), and the morphology of products deliver the polyhedron framework with the size of about 100 nm (**Figure S2**). Meanwhile, according to the TG result of ZIF-8 (**Figure S3**), the decomposition temperature of ZIF-8 begins at 400 ℃ and the reaction is complete around 600 ℃. Thus, this work selected 600 ℃ as the modification temperature.

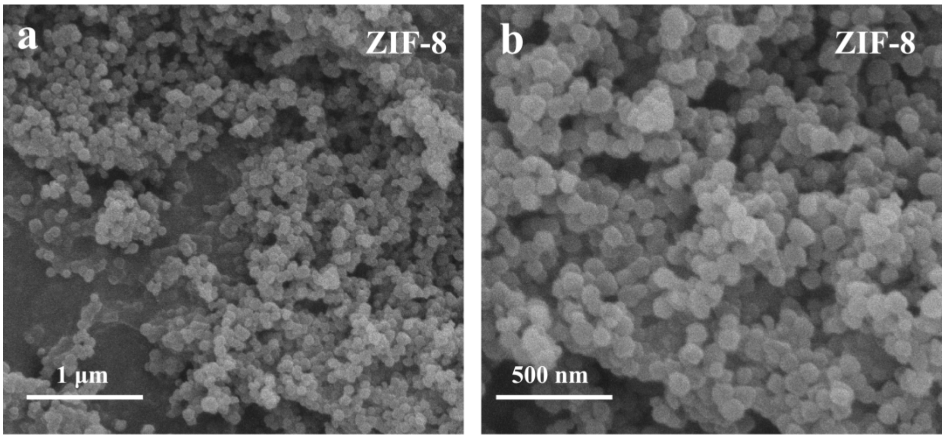

Figure S2. The SEM of ZIF-8.

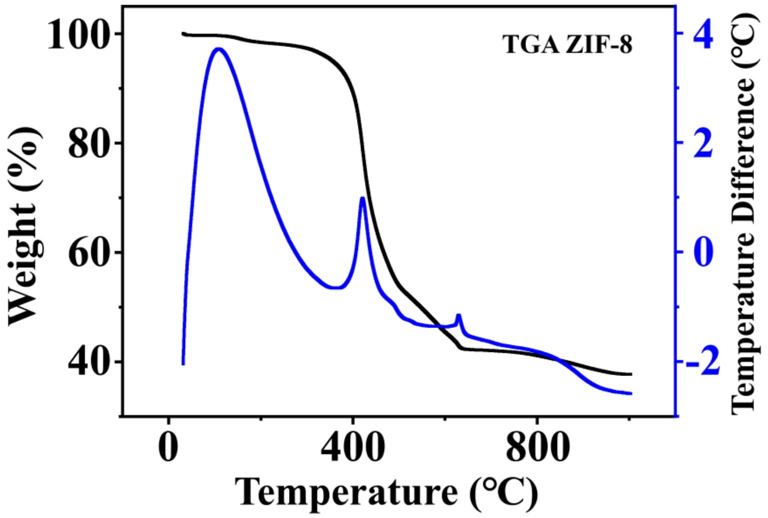

Figure S3. The TGA result of ZIF-8.

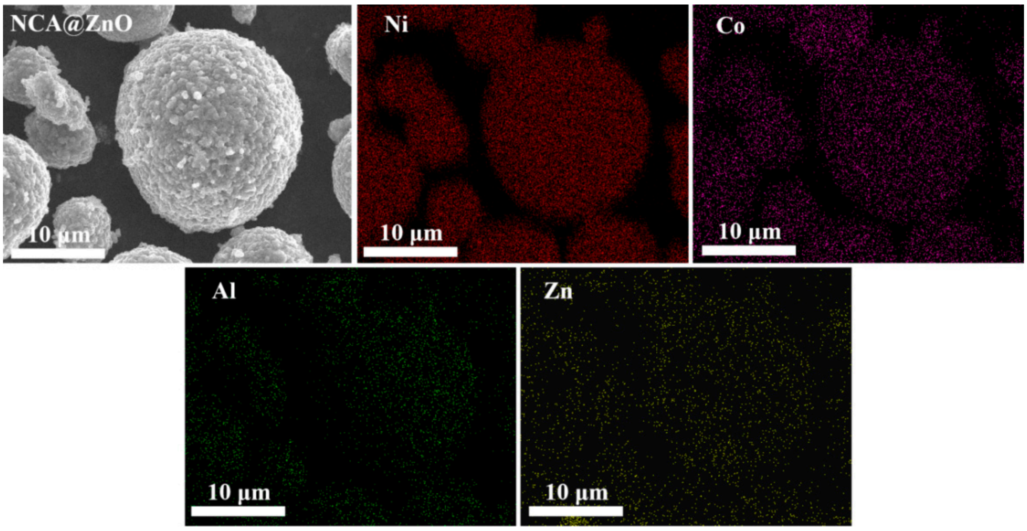

Figure S4. The SEM elemental mapping of NCA@ZnO.

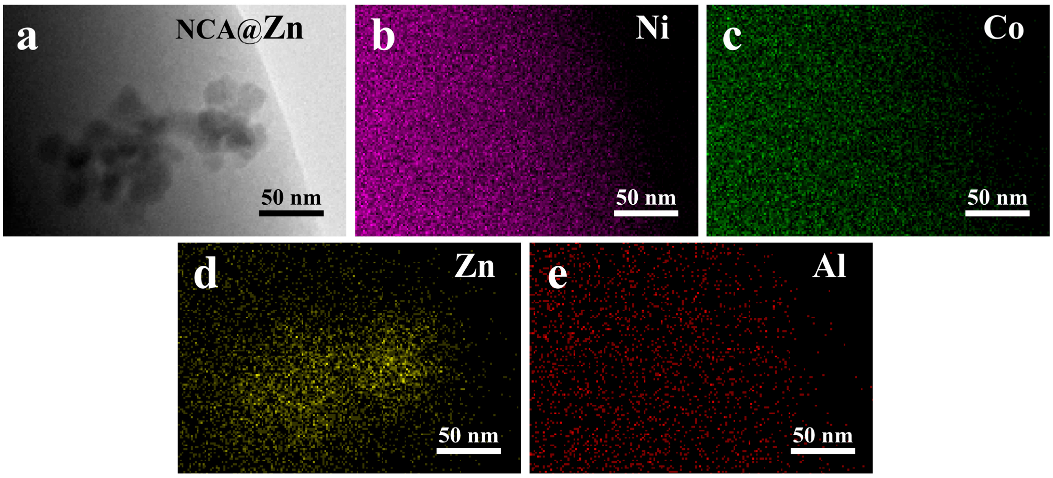

Figure S5. The TEM elemental mapping of NCA@ZnO.

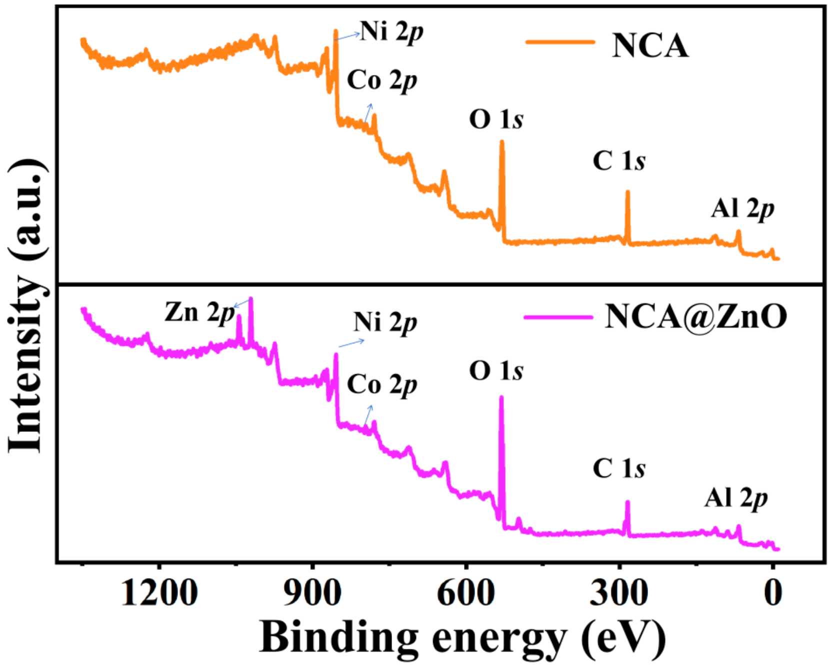

Figure S6. XPS results of NCA@ZnO and NCA.

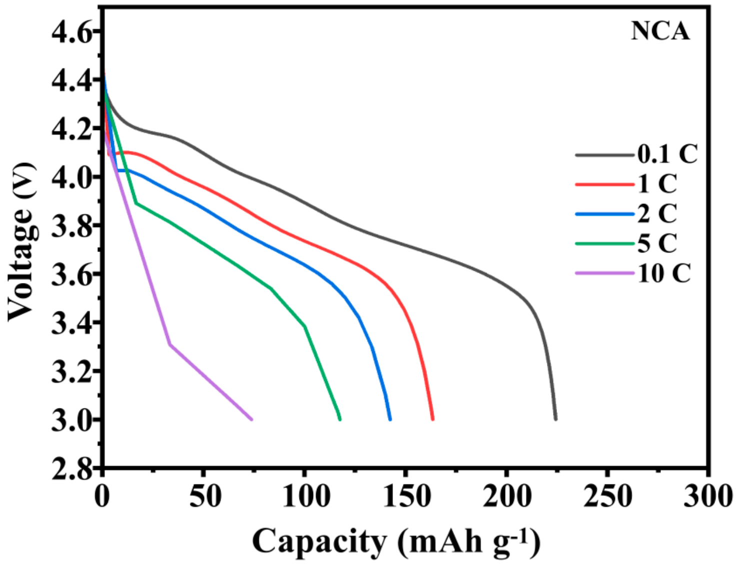

Figure S7. The discharging curves of NCA at various rates.

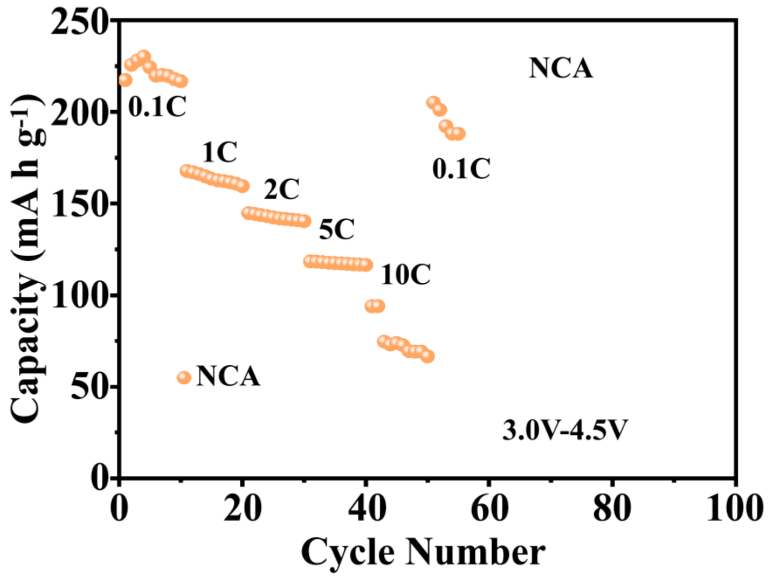

Figure S8. The rate performance of NCA.

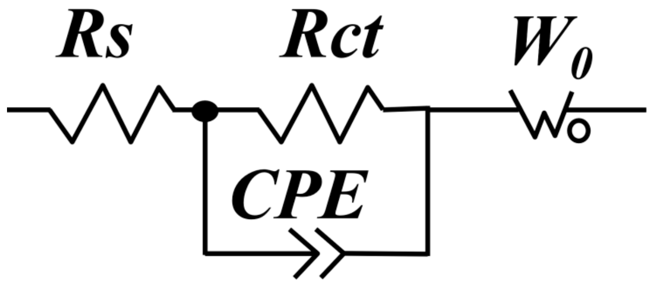

Figure S9. The equivalent circuit for fitting EIS curves of NCA and NCA@ZnO.

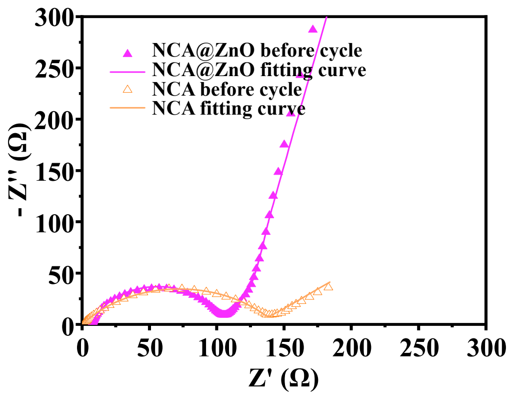

Figure S10. The EIS results of NCA@ZnO and NCA before cycling.

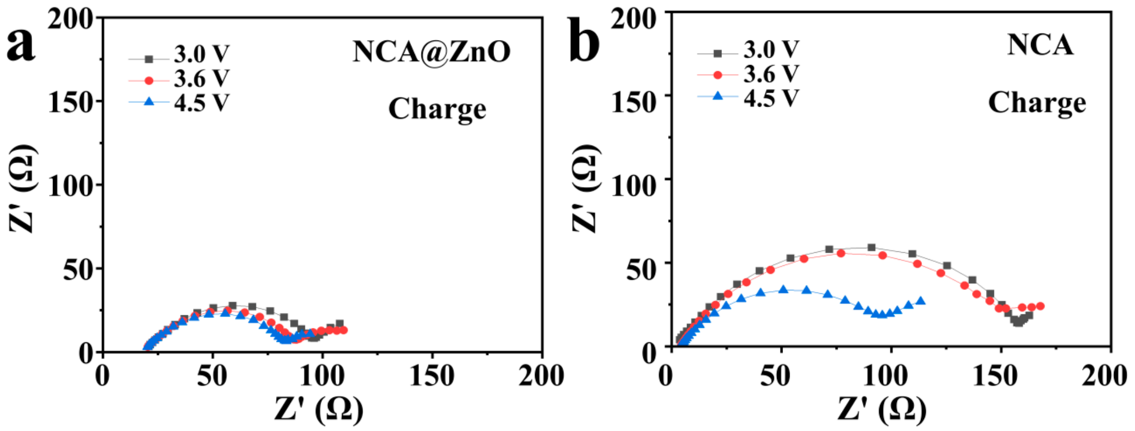

Figure S11. In-siu EIS in charge process for (a, b) NCA@ZnO and c,d) NCA composites, respectively.

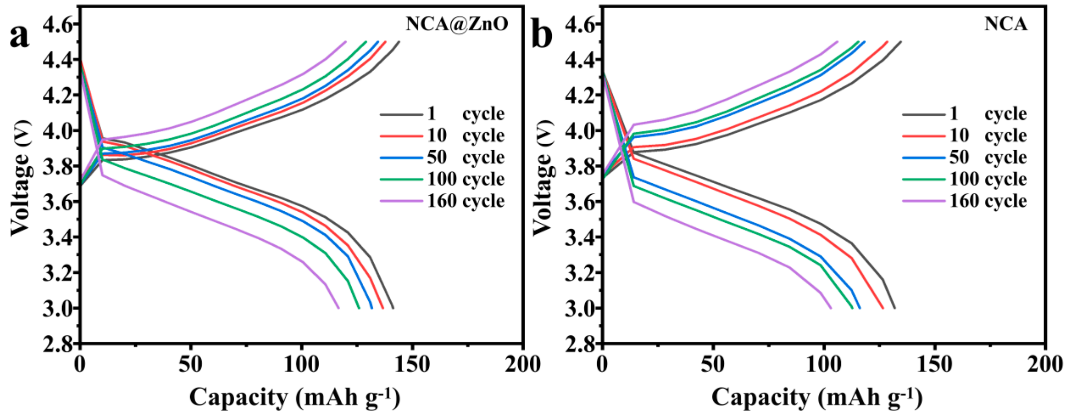

Figure S12. The discharging/charging curves of NCA@ZnO (a) and NCA (b) at various cycles.

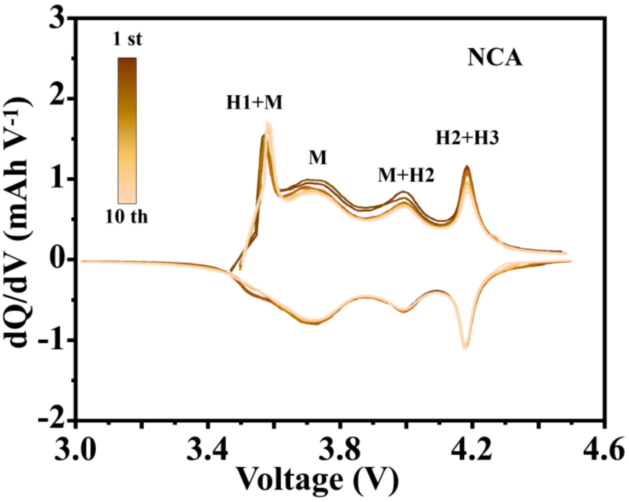

Figure S13. The dQ/dV curves of NCA with different cycling numbers.

Table S1. The refined lattice parameters, c/a and Li/Ni mixing results for NCA@ZnO. and NCA.

Sample	a(Å)	b(Å)	c(Å)	c/a	Li^+/Ni^{2+}
NCA@ZnO	2.866238	2.866238	14.173655	4.945037	3.8%
NCA	2.879044	2.879044	14.212893	4.936670	4.2%

Table S2. The R_{ct} results of NCA and NCA@ZnO in the equivalent circuit.

Sample	NCA@Zn(Ω)	NCA(Ω)
Before cycle	97.5	143.5
After cycle	186.5	224.8

Table S3. The σ_ω values of NCA and NCA@ZnO before and after cycling.

Sample	NCA@ZnO	NCA
Before cycle	1.61	1.80
After cycle	3.04	4.89

Table S4. The D_{Li+} results of NCA and NCA@ZnO in before and after cycle.

Sample	NCA@ZnO(cm^2 s^{-1})	NCA(cm^2 s^{-1})
Before cycle	1.84×10^{-11}	1.48×10^{-11}
After cycle	5.17×10^{-12}	2.01×10^{-12}

To further investigate the diffusion process, we fitted the pre- and post-cycling EIS data using Software ZView to obtain Parameter σ_ω (**Table S3**). The lithium-ion diffusion coefficient (**Table S4**) was then calculated using the following equation.

$$D = R^2T^2/2A^2n^4F^4C^2(\sigma_\omega)^2$$

$$Z' = R_s + R_{ct} + \sigma_\omega + \omega^{-1/2}$$

Where *D*: diffusion coefficient, *R*: gas constant, *T*: temperature, *n*: electron number, *F*: Faraday constant, *C*: Li^+ concentration, σ_ω: Warburg coefficient, respective.
